# Basic Microbiome Analysis: Analytical Steps from Sampling to Sequencing

**DOI:** 10.3390/microorganisms14020387

**Published:** 2026-02-06

**Authors:** Gülfem Ece, Ahmet Aktaş, Özlem Koyuncu Özyurt, Hadiye Demirbakan, Hikmet Eda Alışkan, İmran Sağlık, Orçun Zorbozan, Alev Çetin Duran, Ayşe Rüveyda Uğur, Duygu Öcal, Emel Uzunoğlu, Esra Kaya, Fatma Mutlu Sarıgüzel, Fulya Bayındır, Gülay Yetkin, Mustafa Altındiş, Sevinç Yenice Aktaş, Tuba Kula Atik

**Affiliations:** 1 Department of Medical Microbiology, İzmir City Hospital, İzmir 35540, Türkiye; gulfem.ece@gmail.com (G.E.); f_bilman@hotmail.com (F.B.); 2 İstanbul Public Health Laboratory No. 2, İstanbul Provincial Health Directorate, İstanbul 34524, Türkiye; 3 Department of Medical Microbiology, Faculty of Medicine, Akdeniz Univertsity, Antalya 07070, Türkiye; ozlemozyurt@akdeniz.edu.tr; 4 Department of Medical Microbiology, Faculty of Medicine, Sanko University, Gaziantep 27090, Türkiye; demirbakan78@yahoo.com; 5 Department of Microbiology, Adana Faculty of Medicine, Health Science University, Adana 01790, Türkiye; ealiskan@hotmail.com; 6 Department of Medical Microbiology, Faculty of Medicine, Uludag University, Bursa 16059, Türkiye; imransaglik@uludag.edu.tr; 7 Department of Medical Microbiology, Faculty of Medicine, Bakırçay University, İzmir 35665, Türkiye; orcun.zorbozan@bakircay.edu.tr; 8 Department of Medical Microbiology, Faculty of Medicine, Balıkesir University, Balıkesir 10100, Türkiye; alevctndrn@gmail.com (A.Ç.D.); tkulaatik@gmail.com (T.K.A.); 9 Department of Medical Microbiology, Konya City Hospital, Konya 42020, Türkiye; ayserugur@gmail.com; 10 Department of Medical Microbiology, Faculty of Medicine, Ankara University, Ankara 06230, Türkiye; docal@ankara.edu.tr; 11 Department of Medical Microbiology, Faculty of Medicine, Giresun University, Giresun 28200, Türkiye; emel.uzunoglu@giresun.edu.tr; 12 Department of Medical Microbiology, Kahramanmaraş Necip Fazıl City Hospital, Kahramanmaraş 46100, Türkiye; esra_ytn@hotmail.com; 13 Department of Medical Microbiology, Faculty of Medicine, Erciyes University, Kayseri 38030, Türkiye; fmutluguzel@gmail.com; 14 Bakırköy Dr Sadi Konuk Education and Research Hospital, Hamidiye Faculty of Medicine, Health Science University, İstanbul 34140, Türkiye; gulayyetkin03@gmail.com; 15 Department of Medical Microbiology, Faculty of Medicine, Sakarya University, Sakarya 54290, Türkiye; maltindis@sakarya.edu.tr; 16 Department of Medical Microbiology, Faculty of Medicine, Onsekiz Mart University, Çanakkale 17020, Türkiye; sevincyenice@gmail.com

**Keywords:** metagenomics, human microbiome, bioinformatics pipelines, shotgun metagenomics, machine learning, computational microbiology

## Abstract

The human microbiome is increasingly recognized as a key determinant of health and disease, yet methodological variability continues to limit reproducibility and clinical translation of findings. This review synthesizes current approaches in microbiome research, critically evaluating each step from sampling to sequencing and downstream bioinformatics. Pre-analytical factors such as sample type, collection method, preservation, and storage conditions profoundly affect microbial community profiles and remain a major source of bias. Nucleic acid extraction protocols and quality assessment strategies are discussed with emphasis on optimized lysis techniques, contamination controls, and DNA yield evaluation. Advances in sequencing technologies are highlighted, including 16S rRNA amplicon sequencing, shotgun metagenomics, third-generation long-read platforms, and emerging single-cell and minimal-input methods, each with specific advantages and limitations in taxonomic and functional resolution. Bioinformatics pipelines for taxonomic profiling, variant detection, phylogenetic inference, and functional annotation are compared, with attention to widely used reference databases such as RefSeq, GTDB, and SILVA. Integrative multi-omics approaches, including metatranscriptomics, metabolomics, and genome-scale metabolic modeling, are presented as powerful tools for linking microbial community structure to host physiology and disease mechanisms. Despite these advances, the lack of standardized workflows across pre-analytical, sequencing, and computational steps continues to hinder inter-study comparability and biomarker validation. This review aims to provide a methodological framework that highlights both strengths and limitations of current technologies while underlining the need for harmonized protocols to ensure reproducibility and accelerate the translation of microbiome research into clinical practice.

## 1. Introduction

The human microbiome is commonly used to describe the microorganisms (bacteria, archaea, viruses, and fungi) that inhabit multiple anatomical sites, such as the skin, gastrointestinal tract, oral cavity, respiratory tract, and genitourinary system, in conjunction with the broader biological context in which they function. These microbial populations, referred to as the microbiota, are often used interchangeably with the term microbiome; however, a conceptual distinction is warranted. While “microbiota” denotes the living organisms, “microbiome” encompasses their genomes, metabolites, structural components, and environmental context [1,2]. Using this vocabulary consistently also supports more transparent reporting and study comparability across the field [1,3]. In this review, we use these terms consistently because the distinction becomes critical when moving from “who is there?” to “what can they do?” and “what does this mean for health and disease?”

The human gut harbors one of the most complex and densely populated microbial ecosystems in the body. Large-scale metagenomic sequencing has established extensive catalogs of gut microbial genes, highlighting both the diversity and functional capacity of this ecosystem [4]. This community contributes to key host processes, including fermentation of indigestible polysaccharides, vitamin biosynthesis, maintenance of epithelial barrier integrity, and immune modulation [5]. Disruptions of this ecological balance, termed ‘dysbiosis’, have been associated with various chronic conditions, including metabolic syndrome, neurodevelopmental disorders, and inflammatory bowel disease [5]. Importantly, these associations are often context-dependent and should not be interpreted as simple, direct causality; robust methodology and clinical context are essential for interpretation [3,5].

Technological advances have transformed microbiome science from taxonomic surveys to more functional and clinically oriented insights. In particular, genome-resolved metagenomics has enabled reconstruction of metagenome-assembled genomes (MAGs), supporting strain- and genome-level interpretation and accelerating microbiome medicine research [6]. Beyond genomics alone, multi-omics integration (e.g., combining metagenomics with transcriptomics, metabolomics, and proteomics) is increasingly used to link microbial features to host phenotypes and disease-relevant modules [7,8].

Despite substantial advances, microbiome research faces significant methodological challenges that complicate the interpretation, reproducibility, and clinical translation of findings. Technical variability in sample collection protocols, nucleic acid extraction, library preparation, and sequencing can introduce significant bias [3,5]. Equally important, analytical choices can significantly impact conclusions: differences in taxonomic classification tools and reference databases can substantially alter results, so pipeline selection and reporting must be transparent and standardized [3,6]. This is precisely why reporting and harmonization frameworks (e.g., STORMS and current standardization actions) have become central for improving comparability across studies and enabling clinically meaningful biomarkers [1,3].

This review aims to synthesize current microbiome research methodologies by critically examining each analytical step—from sampling and pre-analytics to sequencing and downstream bioinformatics—while providing a comparative overview of available techniques, including their strengths, limitations, and applications. Although microbiome research has expanded rapidly, progress toward clinical translation is still constrained by methodological heterogeneity, incomplete standardization of analytical workflows, and limited reproducibility across studies [1,3]. To address these unmet needs, we integrate recent advances in sequencing technologies, genome-resolved metagenomics (including MAG reconstruction), and functional profiling with a practical focus on quality control and transparent reporting [1,3,6]. We also highlight how integrative multi-omics strategies can strengthen biological interpretation, improve robustness of findings, and ultimately support clinical translation [3,7,8].

We first discuss pre-analytical steps and significant sources of bias; then, we compare sequencing options and genome-resolved approaches. Next, we summarize key bioinformatic choices and reporting/standardization requirements. Finally, we outline how multi-omics integration can connect microbial signals to disease-relevant phenotypes and translational applications [1,3,6,7,8].

In doing so, this review fills key gaps in the existing literature by offering an end-to-end framework that (i) covers the whole workflow from study design to interpretation, (ii) synthesizes recent technological developments in sequencing and genome reconstruction, and (iii) places technical pitfalls, quality control, and reproducibility at the center of methodological decision making. Through this review, we aim to support both newcomers and experienced researchers while promoting transparency, comparability, and long-term reliability in microbiome research.

## 2. Sample Collection and Pre-Analytical Variables

In microbiome research, ensuring the standardization of pre-analytical processes is essential for reducing the high variability observed, increasing DNA yield, accurately identifying microbial taxa, and obtaining reliable and comparable results. However, clearly defined pre-analytical guidelines have not yet been established in microbiome studies. Existing research in this area may serve as a valuable reference. Critical steps in the pre-analytical process include the development of an appropriate study design, the proper selection of participants, the determination of sample type and collection methods, and the control of sample transport and storage conditions.

Stool sampling is considered the gold standard for gastrointestinal microbiome analysis; however, in situations where stool samples cannot be obtained—particularly in intensive care unit patients—alternative non-invasive methods, such as rectal swabs and glove-tip sampling, are important due to their accessibility. Rode et al. [9] investigated whether the gut microbiota differs across samples collected from different regions of the intestine and included rectal swab samples in their analysis. After collection, samples were placed in DNA/RNA Shield and stored frozen. Their results demonstrated that the microbiota composition of all sample types was highly similar in terms of relative abundance, with Bacillota and Bacteroidota identified as the dominant phyla [10]. In another study, a comparative analysis of stool samples and rectal swab samples collected using E-Swab methods demonstrated that no significant differences in alpha diversity were observed between the two sampling methods. However, samples stored at room temperature exhibited a significant increase in *Escherichia coli* abundance, whereas a smaller increase was observed in *Enterococcus* spp. In contrast, no differences were detected in samples stored at 4 °C [11]. Short-chain fatty acids (SCFAs), which play a critical role in maintaining intestinal barrier integrity, are metabolites exclusively produced by resident gut bacteria. These metabolites have been shown to be associated with dysbiosis and a range of inflammatory disorders. Therefore, the investigation of SCFAs is an important component of stool-based microbiome studies [12,13].

In microbiome studies, to accurately assess gut microbiota diversity and metabolite production, participant-related factors should be systematically evaluated and documented. These include age (as microbial diversity may decrease in older individuals), sex, genetic variations, immune status (with immunosuppressed individuals potentially exhibiting increased dominance of pathogenic taxa), dietary patterns (fiber-rich diets are associated with increased short-chain fatty acid production, whereas diets high in fat and sugar have been linked to dysbiosis and an increased abundance of antibiotic resistance genes), geographic location, hygiene conditions, drinking water quality, and lifestyle factors [14,15].

Sampling time, as an independent variable, can significantly influence microbiome composition. Factors such as circadian rhythms, food intake, and gastrointestinal motility may induce diurnal variations in microbial communities. Significant differences in microbial profiles have been reported in stool samples collected at different times of the day. It is therefore recommended that the first complete bowel movement of the day be collected and that the sample be frozen immediately after collection [12]. During stool collection, urine contamination may occur due to physiological factors. Although smart toilet systems capable of separating urine from feces are currently available, their accessibility remains limited; therefore, commercial stool collection kits are commonly used. Furthermore, stool consistency (hard, soft, or watery) and intestinal transit time may act as selective forces influencing bacterial growth rates and are strongly associated with all major known microbiome biomarkers [16,17]. Vandeputte et al. [18] demonstrated that liquefied stool samples exhibit markedly reduced species richness. The main sample collection methods used in gastrointestinal microbiota research, along with their respective advantages and limitations, are summarized in Table 1.

After sample collection, aliquoting is recommended to prevent DNA degradation caused by repeated freeze–thaw cycles. Prior to aliquoting, homogenization should be performed under anaerobic conditions to minimize the loss of obligate anaerobic bacteria. In particular, stool homogenization is critical for metabolomic analyses. During colonic transit, fecal material is exposed to the mucus layer secreted by epithelial cells, leading to an uneven spatial distribution of microbial taxa across the stool surface [19]. In one study, the inner core of stool samples was shown to harbor significantly higher abundances of Bacillota and *Bifidobacterium* compared with the outer layer, whereas fungal taxa (*Saccharomycetes*) were reported to be reduced. Additionally, differences in aerobic and anaerobic microbial ratios between the outer and inner regions of stool were identified, likely due to oxygen concentration gradients [16,20]. Due to the inherent heterogeneity of stool samples, appropriate homogenization should be performed as the initial step following sample collection. For homogenization, methods such as manual mixing, grinding under liquid nitrogen, or bead-beating techniques may be employed. Carrillo et al. [21] investigated the effects of solvent addition, bead size, and sample lyophilization prior to homogenization on the total number of detected peaks and overall analyte signal intensity. Their findings demonstrated that the optimal homogenization approach, in terms of metabolite abundance and reproducibility, was achieved by using a combination of large and small beads together with organic solvents in wet-frozen stool samples.

Another critical step in the pre-analytical workflow is the transfer and storage of samples prior to analysis. At this stage, transport duration, ambient temperature, the type of preservatives used, and freezing strategies can substantially alter microbiota composition. To prevent microbial DNA degradation and avoid artificial shifts in the distribution of viable taxa, these procedures must be carefully controlled. A study comparing seven different sample collection and storage methods demonstrated that preservative-containing approaches, including RNAlater, fecal occult blood test (FOBT), and fecal immunochemical test (FIT) tubes, largely preserved microbial profiles even after two years of storage at −80 °C [22]. However, it has been reported that while buffers such as RNAlater effectively preserve microbial DNA, they may markedly reduce cell viability and thereby limit subsequent culture-based analyses [16]. Although FOBT and FIT tubes are suitable for clinical screening studies, they are not considered optimal sample collection methods for microbiome research. In contrast, preservatives such as OMNIgene GUT have been shown to exert minimal effects on microbiota composition, whereas ethanol-based collection tubes may compromise microbiota stability [19]. The impact of cryoprotectant use during sample storage on bacterial viability has not yet been fully elucidated, and no consensus has been reached regarding their routine application. Although cryoprotectants may preserve cellular viability during freezing, they can create a nutrient-rich environment for microorganisms upon thawing, potentially promoting microbial proliferation and thereby altering the original microbiota composition [16]. Tedjo et al. [23] reported that no differences in microbial community composition were observed between samples frozen directly at −80 °C without the use of a buffer and those stored at −20 °C for 24 h, or at 4 °C or room temperature for 24 h. If stool samples are to be processed without delay, they should be kept at room temperature for no longer than 4 h or at 4 °C for up to 24 h. For short-term storage of several months, samples should be stored at −20 °C, whereas −80 °C is recommended for long-term storage [16,23]. However, during long-term storage at −80 °C, repeated freeze–thaw cycles should be limited to no more than three cycles, or samples should be aliquoted to avoid repeated freezing and thawing [20]. The major pre-analytical variables that may influence gastrointestinal microbiota composition and data reliability are summarized in Table 2.

In skin microbiome research, numerous factors influence the preanalytical phase. One of the principal determinants affecting microbiome analysis is the selection of the skin site to be sampled [24]. Therefore, during the study design stage, the skin regions to be included in the research should be clearly defined and applied in a standardized manner across all participants. The sebaceous, moist, or dry characteristics of the selected skin sites directly influence microbial composition. For instance, lipid-utilizing *Cutibacterium* spp. and *Corynebacterium minutissimum* are more frequently detected in sebaceous areas, whereas *Corynebacterium* spp. and *Staphylococcus* spp. associated with body odor are more predominant in moist regions. Dry skin sites, although generally characterized by lower microbial biomass, tend to exhibit higher microbial diversity [25].

In studies investigating the relationship between the skin microbiome and a specific disease, the disease stage at which sampling is performed should be determined in advance and applied consistently across all participants. Participant-related factors such as age, sex, ethnicity, personal hygiene practices, and underlying conditions, including diabetes mellitus, may also influence the skin microbiome [26]. Regardless of the anatomical site, it should be taken into account that the relative abundance of *Lactobacillus* spp. and *Cutibacterium* spp. may decrease with advancing age [27]. Since the use of soaps, antiseptics, cosmetic products, or topical agents prior to sampling can alter the microbial profile, it is essential that such practices be documented in detail [24,26].

In the subsequent stage, the sampling method to be used should be determined. In studies focusing on the superficial skin microbiome, surface swab sampling is the most commonly preferred method. The widespread use of this technique offers several advantages, including the availability of a wide range of commercial kits and a relatively standardized approach. Moreover, its frequent use in the literature ensures high comparability of the generated data with those of other studies [28]. For the investigation of the deeper epidermal microbiome, including pores and skin appendages, tape stripping or skin scraping methods are considered more appropriate. It should be taken into account that tape stripping may result in the detection of aerobic bacteria at higher proportions. In cases where dermal skin diseases or the dermal microbiome are being investigated, the punch biopsy method can provide more detailed information; however, due to its invasive nature, its applicability is limited, and achieving an adequate sample size may be more challenging. The combined use of multiple sampling methods may contribute to the identification of a broader taxonomic diversity [24]. The steps to be followed in skin microbiome research are presented in Table 3.

Sampling methodology is of critical importance in urinary microbiome research, as inappropriate sampling may lead to contamination and misinterpretation of microbial profiles. Midstream urine samples primarily provide information on the urogenital microbiome, as they inevitably come into contact with the vulvovaginal microbiota in women and the urethral microbiota in men, thereby increasing the risk of contamination from adjacent microbial niches. In studies specifically aiming to investigate the bladder microbiota, transurethral catheterization or suprapubic aspiration methods are therefore preferred, as these approaches minimize contamination from the distal urogenital tract [29].

**Figure 1:**
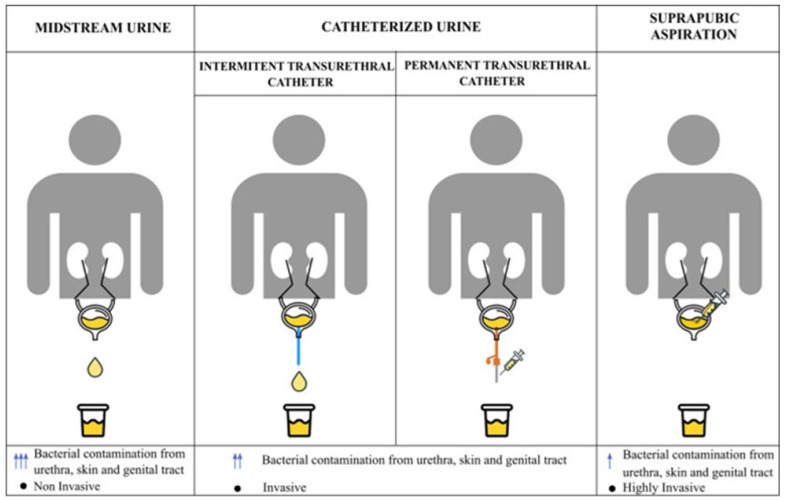
Sampling methods in urinary microbiome research. (Reproduced from ref. [30]).

Current evidence suggests that the microbial profiles obtained using transurethral catheterization and suprapubic aspiration are largely comparable. Given its less invasive nature and greater feasibility in clinical practice, transurethral catheterization is more commonly employed [30]. Regardless of the sampling technique used, strict adherence to standardized preanalytical protocols and detailed documentation of sampling procedures are essential to ensure data reliability and comparability across studies [31]. The sampling methods applicable in urinary microbiome research are illustrated in Figure 1.

Sample storage conditions constitute a critical preanalytical factor that directly influences the outcomes of urinary microbiome analyses. It is recommended that specimens be frozen at −80 °C immediately after collection. When immediate freezing is not feasible, samples may be temporarily stored at +4 °C or −20 °C before transfer to −80 °C; however, in situations where samples must be kept at room temperature, the use of stabilization agents is strongly recommended [32].

Multiple variables influence the preanalytical phase of vaginal microbiome research. Hormonal fluctuations throughout a woman’s life directly affect the composition of the vaginal microbiota. Factors such as life stages, pregnancy, contraceptive use, and sexual activity may lead to alterations in the vaginal microbiome; therefore, the timing of sampling represents one of the most critical preanalytical factors. Periods characterized by elevated estrogen levels are associated with increased *Lactobacillus* dominance, and the vaginal microbiome has been reported to remain relatively more stable during pregnancy. In addition, smoking, hygiene practices, and dietary habits may also influence the vaginal microbiome. These variables should be carefully considered during study design, and inclusion criteria should be clearly defined. Vaginal swab samples are most commonly used in vaginal microbiome analyses, and efforts should be made to minimize the risk of urinary or rectal contamination during sampling [33].

Respiratory microbiome research is generally classified into studies focusing on the upper and lower respiratory tracts. In investigations of the upper respiratory tract microbiome, swab samples collected from the nasal cavity or nasopharynx using sterile swabs are commonly employed [17]. Non-invasive methods, such as sputum and tracheal aspirates, can provide microbial information related to both the upper and lower respiratory tracts and offer advantages due to their ease of application and repeatability. Moreover, these sample types typically contain a higher microbial biomass compared to bronchoalveolar lavage samples. However, the anatomical region of the respiratory system represented by data obtained through these methods cannot always be clearly delineated [34]. A recent study reported no significant differences between bronchoalveolar lavage and tracheal aspirate samples in the assessment of the lung microbiome [35].

In lung microbiome studies, the generally accepted sampling methods include bronchoalveolar lavage, protected brush sampling, and lung biopsy. Nevertheless, due to their invasive nature, these approaches present limitations in terms of feasibility and repeatability, making it more challenging to achieve adequate sample sizes. Unlike other sampling methods, lung biopsy provides microbial information that is specific to the lung parenchyma [34]. The sampling methods used in respiratory microbiome research are summarized in Table 4.

In the majority of non-GIS microbiome studies, the low microbial biomass of sampled sites substantially increases the risk of contamination during both preanalytical and analytical processes. Contamination may arise from the external environment, laboratory reagents, consumables, or host-derived DNA/RNA sources, potentially leading to biased or misleading results. Therefore, strict adherence to aseptic conditions throughout all stages—from sample collection to laboratory processing—is essential. Appropriate hygiene practices should be implemented prior to sampling, and contamination control strategies should be systematically incorporated into the analytical workflow. These strategies include the use of negative controls (e.g., blank sampling and extraction controls) to identify background contamination, as well as positive controls to monitor methodological consistency and analytical performance. The rigorous application of these measures is critical to ensure the reliability, reproducibility, and interpretability of microbiome data derived from low-biomass samples [17,36].

## 3. Nucleic Acid Extraction and Quality Assessment

### 3.1. Sample-Dependent Challenges and Lysis Strategies

The process of nucleic acid extraction is critical for the accuracy and reproducibility of microbiome analyses. Thus, the standardization of extraction techniques and stringent quality evaluation have become vital to satisfy the escalating need for rapid, reproducible, and high-throughput microbial profiling.

Microbial habitats vary significantly in microbial load, taxonomic composition, and matrix-associated variables, including salt and protein concentrations, the presence of PCR inhibitors, and host-derived nucleic acids. Samples exhibiting low microbial biomass (10^3^–10^4^ CFU/mL), such as neonatal stool or tissue-associated microbiomes, frequently contain a substantial amount of human DNA and are especially prone to background contamination, which can significantly affect subsequent sequencing results [17]. Eliminating host DNA can be addressed by enhancing microbial DNA through a differential lysis step [37]. Variations in bacterial cell wall architecture markedly influence lysis efficiency. Gram-positive bacteria and mycobacteria, distinguished by their thick peptidoglycan layers and, in the case of mycobacteria, mycolic acid-enriched outer membranes, exhibit greater resistance to chemical and enzymatic lysis compared to Gram-negative bacteria. In addition to bacteria, organisms possessing structurally resilient cell structures, such as spore-forming bacteria and fungi with chitinous cell walls, also frequently require vigorous mechanical or coupled mechanical-chemical lysis procedures to guarantee successful nucleic acid extraction [37]. Consequently, extraction methods relying exclusively on chemical or enzymatic lysis may underestimate these species, thus creating systematic bias in microbiome profiles [38]. Mechanical lysis techniques, notably bead beating, have demonstrated efficacy in enhancing DNA recovery from structurally resilient microorganisms and augmenting observed microbial diversity, particularly the relative prevalence of Gram-positive taxa such as Bacillota and Actinomycetota in complex matrices such as feces [38]. Excessive mechanical disruption may result in DNA shearing, which could impact applications necessitating long-read sequencing applications. Conversely, milder chemical or enzymatic lysis techniques often maintain DNA integrity but may preferentially recover easily lysed Gram-negative bacteria, leading to diminished overall diversity estimates [16].

### 3.2. Methodological Bias and Benchmarking Evidence

Benchmarking studies comparing extraction techniques have repeatedly shown that the selection of lysis strategy significantly affects both alpha diversity analyses and taxonomic composition. Therefore, no single extraction technique is consistently optimal. The choice of nucleic acid extraction procedures should be determined by sample type, microbial biomass, target taxa, and subsequent analytical requirements. Thus, each sample type—such as saliva, feces, lung tissue, urine, breast milk, skin, vaginal smear, and oral swab—requires specific methodological approaches for cell lysis, nucleic acid extraction, and purification processes [39]. Additionally, different commercial kits may provide divergent DNA extraction efficiency for taxa, based upon the specific kit employed [40]. Thorough evaluation of these aspects is crucial to reduce methodological bias and ensure biologically valid assessment of the microbial data. The implementation of mechanical and/or chemical lysis methods for nucleic acid extraction has been revealed to affect the relative abundance of certain microbial taxa, suggesting differences in lysis performance among microbial species. The utilization of pre-treatment steps might improve total nucleic acid extraction; yet, several investigations have shown that these methods do not inherently improve overall microbial diversity [41]. Furthermore, enhancements in total nucleic acid yield do not necessarily result in better biological representation of microbial communities [42]. In tissue-associated specimens as lung tissue, pre-treatment steps have been shown to selectively remove extracellular DNA from dead bacteria, thereby improving the accuracy of the microbial composition to a true representation [41]. Similar to lung tissue, urine exhibits a comparatively low microbial biomass, making it crucial to carefully consider the representativeness of each bacterium to ensure reliable microbiome profiling. Commercial nucleic acid isolation kits tested on urine samples provided significantly variable DNA yields, whereas differences in alpha and beta-diversity, and the number of recovered reads were not statistically significant [43]. Table 5 summarizes major lysis strategies on major microbial communities, demonstrating how variations in cell wall structure affect lysis efficiency and potential biases in microbiome profiling. These data collectively highlight that differences in lysis efficacy across microbial species are an important contributor to methodological bias, emphasizing the importance of adapting extraction techniques in tandem with the biological characteristics of the target community.

### 3.3. Quality Assessment: Contamination, Inhibitors, and Purification

The inclusion of appropriate positive and negative controls is essential in microbiome research to assess the accuracy of DNA extraction and identification of potential contamination sources [44]. Positive control samples, which include mock microbial communities with known composition derived from bacterial strains and also from DNA, allow the assessment of extraction efficacy and technique-based biases across different taxa [45]. Conversely, negative controls, including extraction blanks and reagent-only controls, are crucial for identifying environmental contamination introduced during the extraction procedure [17]. Numerous investigations have shown that DNA extraction kits and laboratory chemicals could contain small quantities of bacterial DNA, known as the “kitome,” which can substantially affect microbial profiles, especially in low-biomass samples. Contaminant DNA frequently derives from water, buffers, enzymes, and plasticware, perhaps resulting in the erroneous identification of environmental or skin-associated taxa. Well-to-well contamination, referred to as the “splashome,” constitutes an additional source of inaccuracy in microbiome research with low-biomass samples [46].

To reduce contamination, stringent laboratory protocols are necessary, including the physical segregation of pre- and post-PCR workflows, the use of DNA-free equipment, routine use of negative controls in every extraction session, and the use of personal protective equipment. Supplementary techniques, such as UV treatment of work surfaces and reagents when appropriate, can mitigate exogenous DNA contamination; however, UV irradiation may not eradicate all DNA fragments and should be regarded as a complementary rather than the main strategy [17]. The systematic inclusion of extraction blanks, negative controls, and contamination-aware handling protocols is crucial for differentiating actual biological signals from reagent- or laboratory-induced contamination, thereby enabling accurate assessment of microbiome data [47].

### 3.4. Reproducibility: Extraction Variability and Batch Effects

Extraction-induced batch effects are a significant obstacle to reproducibility in microbiome research, undermining the reliability of cross-study comparability and meta-analyses. Even when utilizing similar specimens, varying DNA extraction procedures might produce significantly varied estimations of microbial biomass and taxonomic profiles owing to variability in lysis efficiency, DNA recovery, and purification processes [48]. This protocol-dependent variability constitutes a significant source of technical batch effects, where discrepancies arising during sample processing obscure inherent biological diversity. Batch effects resulting from extraction variability can cause systematic variations in relative abundance, mislead diversity data, and severely hinder comparability across specimens processed in various batches, facilities, or laboratories [49].

The efficiency and reliability of nucleic acid extraction are crucial factors influencing data quality in microbiome research. Consequently, employing optimized, specific, and sensitive extraction techniques, alongside protocol standardization and consistent handling of samples within and between studies, is crucial for maintaining relative microbial abundances and achieving reproducible, biological results, avoiding inconsistent conclusions and interpretations [48].

The selection of nucleic acid extraction kits should be determined by sample biomass, matrix complexity, and the specific downstream application (DNA or RNA analysis). High-biomass samples, such as adult feces, typically withstand more rigorous lysis techniques and are enhanced by kits designed for extensive microbial coverage. Conversely, low-biomass samples, such as tissue-associated or respiratory specimens, necessitate extraction techniques that reduce background contamination and host DNA interference [50].

### 3.5. Practical Considerations: Kit Selection, Host DNA, RNA Workflows, and Automation

Moreover, microbiome research centered on DNA and RNA includes specific technical specifications. DNA-based procedures emphasize efficient and impartial recovery across various microbial taxa, whereas RNA-based investigations require swift stabilization, rigorous RNase management, and extraction methods tailored for the preservation of unstable RNA molecules. Therefore, no extraction kit is universally applicable; intelligent selection based on sample attributes and research goals is essential to guarantee data quality and repeatability (Table 6) [51].

In samples where host-derived nucleic acids significantly surpass microbial DNA, such as tissue-associated, respiratory, or biopsy specimens, elevated host DNA levels provide a substantial technical obstacle for microbiome studies. Excess host DNA can obscure microbial populations by reducing the proportion of DNA sequence reads from the microbial component, diminishing sequencing efficiency, and impairing taxonomic accuracy, particularly in shotgun metagenomic methodologies [55].

Numerous approaches have been developed to address this issue, including selective lysis of host cells accompanied by microbial DNA preservation, enzymatic degradation of host DNA, and commercial kits for host DNA depletion or microbial enrichment [56]. These methodologies seek to enhance the microbial signal-to-noise ratio by selectively eliminating host-derived nucleic acids before sequencing [57]. Selective depletion approaches may induce biases by disproportionately impacting specific microbial taxa or impairing cell integrity, highlighting the necessity for meticulous validation and implementation of appropriate controls [58,59]. Therefore, techniques for host-DNA reduction should be selected according to sample type, microbial biomass, and downstream analytical targets along with a thorough assessment of their potential effects on microbial community composition [58].

### 3.6. RNA-Based Workflows

Metatranscriptomics is a comprehensive method for identifying all active microorganisms within a host’s immune response milieu. Metatranscriptomics provides extensive insights into active microbial populations and their interactions with the host, necessitating optimized RNA extraction procedures for reliable and impartial analysis [59]. RNA-based microbiome studies present more significant technological challenges than DNA-based techniques [51]. RNA molecules exhibit intrinsic instability and are very susceptible to breakdown by ubiquitous RNases, necessitating immediate sample stabilization, stringent RNase-free handling, and optimized extraction techniques [40].

In addition to RNA instability, co-extracted inhibitory substances such as polysaccharides, bile salts, phenolic compounds, and residual extraction reagents can adversely affect reverse transcription and downstream enzymatic reactions. These inhibitors are particularly problematic in complex matrices such as feces, sputum, or environmental samples [60].

Furthermore, residual genomic DNA can lead to false-positive transcriptional signals that confounds RNA-based analyses. Consequently, DNase treatment is an essential procedure in RNA extraction protocols for ensuring a targeted examination of transcriptionally active microbial communities. Nonetheless, DNase treatment may induce variability or result in RNA loss if not carefully manipulated, underscoring the necessity for proper control and validation protocols. These problems highlight the necessity for particular optimization and quality control methods in RNA extraction protocols, distinct from DNA-based workflows, to ensure precise interpretation of microbial gene expression profiles [61].

Recent comparative benchmarking studies have confirmed that the selection of DNA extraction kits and protocols significantly influences technical variability, impacting DNA yield, inhibitor carryover, and the observed community composition across different kinds of specimens. A summary of comparative benchmarking studies published from 2020 to 2025 that evaluate DNA extraction kits and techniques is included in Table 7. The studies demonstrate that variability associated with extraction can affect microbial community profiles, DNA yield, and contamination levels, highlighting the necessity of technique selection and standardization, especially when comparing results across different research and specimen types.

Alongside extraction efficiency, the existence of co-extracted inhibitory compounds is a pivotal element affecting downstream sequencing quality in microbiome research. Common inhibitors, including bile acids, polysaccharides, humic compounds, and residual extraction reagents, can disrupt enzymatic reactions during PCR amplification, library preparation, and sequencing, consequently resulting in diminished read depth and biased microbiome composition [70]. To reduce such consequences, purifying methods such as silica-based spin columns, magnetic bead clean-up techniques, and inhibitor removal procedures are commonly integrated into extraction workflows [71]. Although these methods enhance nucleic acid purity and sequencing efficacy, excessive purification may lead to DNA loss, especially in low-biomass samples. Thus, the selection and improvement of purification techniques must be methodically optimized to improve sequencing quality while preserving microbial coverage [72].

Automated nucleic acid extraction technologies are widely utilized in extensive microbiome studies owing to their high throughput capability, enhanced repeatability, and diminished hands-on time [72]. Automation reduces operator-dependent variability, enabling consistent processing of extensive sample cohorts, which is especially beneficial in population-based and multi-center investigations. However, automated extraction platforms may present particular constraints, such as diminished independence in protocol customization, varying lysis performance for structurally resilient microorganisms, and an elevated susceptibility to systematic contamination if stringent control protocols are not maintained. Furthermore, cross-sample contamination and reagent-derived background signals may be exacerbated in high-throughput automated workflows, emphasizing the necessity of extraction blanks, plate-based negative controls, and careful process management [63]. Therefore, although automated extraction technologies provide significant advantages in scalability and reproducibility, their implementation necessitates cautious validation and quality control to ensure that increased throughput does not affect microbial representation or sequencing accuracy [73].

Future advancements in microbiome research emphasize the standardization and transparency of nucleic acid extraction protocols, especially for large-scale and multi-center research. The incorporation of automated extraction systems alongside sophisticated lysis methods and low-contaminant solutions might boost reproducibility while keeping substantial biological representation. Simultaneously, the enhanced utilization of standardized mock communities, extraction blanks, and reporting requirements will be essential for promoting comparability between studies. Innovative methodologies, including as sample-specific extraction optimization, host DNA depletion techniques, and workflow benchmarking across sequencing technologies, are expected to significantly contribute to overcoming existing restrictions. These advancements collectively seek to transition the field towards more robust, reproducible, and clinically translatable microbiome analyses.

## 4. Sequencing Technologies for Microbiome Studies

Microbiome research involves five fundamental steps: study design, sampling, sequencing, analysis, and reporting. Among these, the sequencing approach is one of the most critical components, as it significantly influences both taxonomic resolution and biological interpretation. Over the years, sequencing technologies have undergone substantial evolution—from the Sanger method to second-generation amplicon sequencing, shotgun metagenomics, long-read sequencing, and, more recently, minimal-input approaches suitable for single-cell and low-biomass samples [68]. Because platforms differ in read length, throughput, and characteristic error behavior, the same sample can yield different taxonomic and functional outputs depending on the chosen approach [68]. Accordingly, sequencing approach selection should be aligned with study goals (taxonomic profiling, functional inference, or genome reconstruction), sample properties (biomass/complexity), and available resources [68,74] (Table 8).

### 4.1. First-Generation Sequencing: Sanger

The Sanger sequencing method, known for its high accuracy (~99.9%) and read length of approximately 1000 base pairs, was a pioneer in genetic studies. However, due to its low throughput and high cost, its application in microbiome research has been limited. In current microbiome workflows, Sanger is mainly used for targeted confirmation rather than community-scale profiling [68].

### 4.2. Second Generation: Amplicon Sequencing and Error Correction

Amplicon sequencing targeting the 16S rRNA—especially on Illumina platforms—has become widely used in microbiome taxonomy due to its high sample throughput and cost-effectiveness [82,83]. Nevertheless, since it targets only short regions of the gene (e.g., V3–V4), its taxonomic resolution at the species level is often inadequate. Moreover, PCR-based amplification can introduce biases in abundance estimation. Amplicon workflows are also sensitive to primer and variable-region selection (V1–V9), which can shift observed community composition through differential amplification efficiency. Additional limitations include amplification artifacts (e.g., chimeras) and marker-gene copy number variation, which can distort relative abundance estimates even when sequencing quality is high [75].

The DADA2 algorithm, developed in response to these limitations, enables high-resolution identification of biological variants by modeling sequencing errors using a probabilistic error-correction model that infers exact amplicon sequence variants (ASVs) [77]. This improves resolution compared with OTU-based approaches and reduces spurious diversity arising from sequencing/PCR noise, although it does not remove primer/region-driven biases inherent to amplicon designs [75,76].

### 4.3. Shotgun Metagenomic Sequencing

Shotgun metagenomic sequencing enables random sequencing allows for the random sequencing of all microbial DNA in a sample. This workflow provides a broader taxonomic scope, including not only bacteria but also archaea, viruses, and fungi [68,74,77,78]. It also aids in functional analyses by profiling genetic capabilities. However, this workflow typically requires substantial DNA input, produces large datasets, and requires advanced computational infrastructure. The depth and coverage of sequencing are important factors to consider: deeper sequencing generally enhances the detection of low-abundance taxa, stabilizes functional profiles, and improves the completeness of assemblies and genome reconstruction, but at a higher cost and computational workload. In practical terms, low-depth (“shallow”) shotgun sequencing can be beneficial for broad community screening, while deeper shotgun sequencing is typically necessary for robust functional inference or genome-resolved analyses [74].

### 4.4. Long-Read and Real-Time Sequencing

Third-generation sequencing technologies have ushered in a new era for microbiome research. PacBio HiFi (High-Fidelity) technology provides long (15–25 kb) and highly accurate reads, allowing comprehensive analysis of the full 16S rRNA or entire microbial genomes [68,79]. Oxford Nanopore sequencing, with its portability and real-time capabilities, offers practical advantages for field applications [61]. However, the relatively high error rate of Nanopore sequencing and the ongoing development of analysis software remain important considerations [79,81]. Because long reads can span repetitive regions and improve assembly contiguity, long-read (or hybrid) strategies can enhance strain-level resolution and genome reconstruction compared with short-read-only workflows [68,79]. Clinically, Nanopore-based 16S rRNA sequencing has shown promise in the rapid and direct identification of pathogens in sterile body fluids [81]. Hybrid assemblies (combining long reads for structure and short reads for polishing) may be beneficial when both contiguity and base-level accuracy are required for downstream analyses [74].

### 4.5. Single-Cell and Minimal Sequencing Approaches

Single-cell sequencing workflows enable detailed investigation of microbial heterogeneity. Microfluidics-based platforms, such as Microbe-seq, enable strain-level resolution from the DNA of a single cell. Similarly, M3-seq offers single-cell transcriptomic profiling [68]. Minimal sequencing methods, such as 2bRAD-M, can generate species-level taxonomic profiles from as little as 1 picogram of DNA, making them particularly useful for low-biomass or degraded samples. These approaches address situations where traditional workflows are limited by input constraints, including challenging clinical and tissue-associated microbiomes [78].

### 4.6. Clinical Applications and Standardization

The advancement of sequencing technologies has improved the accuracy of both taxonomic and functional microbiome analyses, paving the way for personalized medicine. However, widespread clinical implementation will rely on the development of standardized sequencing protocols, as well as robust sampling and analysis workflows. In this context, sequencing approach choice should be explicit and justified (e.g., amplicon for high-throughput community profiling; shotgun for combined taxonomic–functional profiling; long-read or hybrid strategies when improved assemblies and strain-level resolution are priorities; and minimal-input approaches for low-biomass samples). Standardization across laboratory and analytical steps is crucial to ensure reproducible results and clinically interpretable outputs [68,74,82].

## 5. Bioinformatics and Taxonomic Profiling

Bioinformatics constitutes a pivotal component of microbiome research by enabling the systematic processing, classification, and biological interpretation of complex sequencing datasets derived from high-throughput sequencing [84]. Among molecular-based strategies, metataxonomics and shotgun metagenomics serve distinct yet complementary roles, and their selection should be guided by the required taxonomic resolution, functional depth, and study objectives. Metataxonomic approaches, based on conserved phylogenetic markers such as the 16S rRNA, 18S rRNA, or ITS regions, offer a cost-effective and scalable solution for profiling microbial community composition; however, their reliance on short marker regions inherently limits species- and strain-level resolution and precludes direct functional inference [84].

In contrast, shotgun metagenomic sequencing enables comprehensive genome-wide analysis, facilitating species- and strain-level classification, detection of single-nucleotide polymorphisms (SNPs), and reconstruction of MAGs [85,86]. The accuracy of such analyses is strongly influenced by the choice of reference database. RefSeq provides broad taxonomic coverage and high-quality curated genomes, making it well-suited for species-level classification in clinical and environmental studies [85]. The Genome Taxonomy Database (GTDB) further improves phylogenetic consistency by incorporating MAGs and redefining microbial taxonomy based on genome-wide evolutionary relationships, thereby offering clear advantages for genome-resolved metagenomic analyses [85].

By comparison, Greengenes, while historically important for 16S rRNA-based studies, is now limited by infrequent updates, reducing its applicability for contemporary microbiome research where novel taxa and genome-resolved approaches are increasingly prevalent [87]. For transcriptomic investigations, RNA sequencing enables quantitative assessment of gene expression dynamics, with tools such as STAR for alignment and DESeq2 for differential expression analysis remaining widely adopted due to their statistical robustness and reproducibility [88]. In addition to RNA sequencing, metagenomic analyses allow for the quantitative measurement of microbiome profiles in both clinical and environmental samples [85].

Beyond taxonomic profiling, genomic variation analysis, including SNP detection, provides critical insight into strain-level diversity and evolutionary dynamics within microbial communities [86]. Phylogenetic reconstruction tools such as IQ-TREE 2, PhyML, and RAxML enable inference of evolutionary relationships, with method selection depending on dataset size, model complexity, and computational constraints [89,90,91]. Collectively, these bioinformatic approaches underscore that pipeline and database selection should be driven by analytical goals rather than convention, as inappropriate methodological choices can substantially bias biological interpretation (Table 9).

Molecular-based approaches, particularly metagenomics and metatranscriptomics, are central to elucidating both the functional potential and the active metabolic state of microbial communities [92]. Metagenomics targets the total DNA content of all organisms within a sample, enabling comprehensive identification of community members and characterization of their collective gene repertoire [93,94]. In contrast, metatranscriptomics focuses on RNA molecules transcribed from these genes, thereby providing insights into actively expressed functions under specific environmental or physiological conditions [94]. Although these approaches interrogate different molecular layers, they share similar analytical workflows, including sequence preprocessing, taxonomic classification, and functional annotation, allowing many bioinformatic tools to be adapted across both data types [95].

Taxonomic classification in metagenomic studies relies on a diverse range of computational strategies, each characterized by distinct trade-offs between accuracy, speed, and computational requirements. Alignment-based methods, such as BLAST and DIAMOND, assign taxonomic labels based on sequence similarity to reference databases and are generally associated with high classification accuracy, particularly for well-characterized taxa [96]. However, their substantial computational cost limits scalability for large datasets. In contrast, k-mer–based classifiers, including Kraken and Kraken2, offer ultrafast sequence classification by matching short sequence signatures, making them well suited for large-scale or time-sensitive analyses, albeit with increased sensitivity to database completeness and sequencing errors [97].

Alternative indexing strategies have been developed to mitigate computational constraints. Centrifuge, which employs FM-index–based data structures, significantly reduces memory usage while maintaining competitive classification performance. Kaiju, operating at the protein level, is specifically designed to handle low-complexity and highly divergent sequences, thereby improving taxonomic resolution in metagenomes derived from poorly characterized environments [98]. More specialized tools, such as CommunBugSplit, align metagenomic assemblies against reference databases and have demonstrated improved performance, achieving up to 33% higher F1 scores compared to several commonly used classifiers [99]. Meanwhile, Emu addresses species-level profiling challenges by leveraging full-length 16S rRNA Nanopore sequencing data and applying an expectation–maximization algorithm to refine abundance estimates, offering enhanced resolution in complex microbial communities [100].

Beyond similarity-based approaches, composition-based classification methods exploit intrinsic sequence features such as GC content, oligonucleotide frequencies, and codon usage patterns. These strategies are particularly advantageous in scenarios where reference databases are incomplete or biased. Machine learning–assisted frameworks, including PhyloPythiaS, have demonstrated robust performance under such conditions by integrating compositional features with supervised learning techniques [101]. Hybrid binning algorithms, such as CONCOCT and MetaBAT, further improve classification accuracy by jointly considering sequence composition and coverage information across samples, making them especially effective for MAG reconstruction [102,103]. More recently, deep learning–based approaches, including tools such as Taxometer, have emerged, utilizing tetranucleotide frequency patterns to capture complex sequence signatures and further enhance taxonomic resolution as machine learning methodologies continue to advance [104].

Hybrid classification approaches aim to integrate the complementary strengths of alignment-based and composition-based strategies by jointly exploiting sequence similarity, statistical features, and coverage information to enhance taxonomic assignment accuracy. For instance, MetaPhlAn employs a clade-specific marker gene framework that enables high-resolution, species-level profiling while minimizing false-positive classifications, making it particularly suitable for well-characterized microbial communities [105]. Similarly, MaxBin combines sequence coverage, GC content, and marker gene information to generate more reliable genome bins, thereby improving MAG reconstruction in complex samples [106].

Taxonomic profiling of metagenomic data represents a foundational step in characterizing microbial diversity; however, its accuracy and resolution are strongly contingent upon the quality, completeness, and currency of the underlying reference databases. A diverse range of taxonomic databases has been developed to accommodate different analytical strategies, including genome-based, rRNA-based, protein-based, and marker gene–based frameworks. Consequently, database selection should be guided by both the target organism group (e.g., bacteria, archaea, fungi, or viruses) and the methodological principles of the chosen classification tool. Importantly, the use of up-to-date and well-curated databases substantially reduces misclassification rates and improves the biological interpretability of metagenomic analyses.

Among genome-centric resources, RefSeq, curated and maintained by NCBI, provides high-quality genomic, transcriptomic, and protein sequences with broad taxonomic coverage, making it a robust reference for species-level genomic and clinical metagenomic studies [107]. In contrast, the Genome Taxonomy Database (GTDB) offers a phylogenetically consistent taxonomy derived from whole-genome data and incorporates a large number of MAGs, thereby addressing limitations of traditional taxonomy based on phenotypic or partial sequence information [108]. GTDB is commonly used in conjunction with tools such as GTDB-Tk and is increasingly favored in genome-resolved metagenomic workflows where evolutionary consistency is prioritized over historical nomenclature.

For amplicon-based studies, SILVA remains a widely adopted database due to its high-quality aligned rRNA sequences, comprehensive phylogenetic frameworks, and regular updates, supporting reliable taxonomic assignment in 16S and 18S rRNA gene analyses [109]. By contrast, Greengenes, despite its historical importance, has become less suitable for contemporary microbiome studies owing to infrequent updates and limited incorporation of newly described taxa [87]. Marker gene–based databases underpinning tools such as MetaPhlAn enable precise species-level identification and have been successfully integrated into functional profiling pipelines such as HUMAnN, facilitating joint taxonomic and functional inference [110].

**Figure 2:**
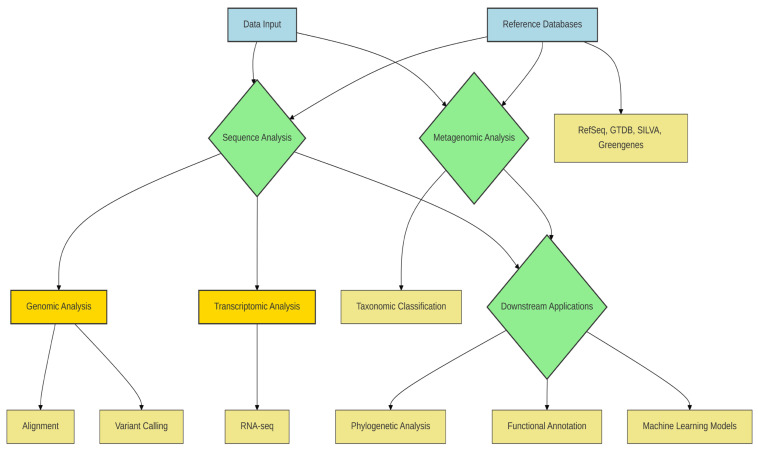
Workflow of microbiome data processing and downstream analytical approaches.

Protein-level classification tools, including Kaiju, typically rely on comprehensive protein databases such as RefSeq or the non-redundant (NR) database and are particularly effective for classifying short, divergent, or low-complexity sequences that are challenging for nucleotide-based approaches [98]. Finally, integrated platforms such as MGnify (EMBL-EBI) provide end-to-end support for both taxonomic and functional analyses of environmentally derived metagenomes, offering standardized pipelines and public data integration to enhance reproducibility and cross-study comparability [111] (Figure 2) (Table 10).

## 6. Functional and Metabolic Inference

A large proportion of microbiome studies focus on the taxonomic composition of microbial communities, which is most commonly inferred from 16S rRNA sequencing data [78]. However, taxonomic information alone provides limited insight into the actual biological activity of microbial communities and their interactions with the host. Comparative analyses of metagenomic and metatranscriptomic data have demonstrated that gene presence does not necessarily correspond to gene expression or metabolic activity [54]. Therefore, the extraction of functional and metabolic features from microbiome sequencing data is critical for a more comprehensive understanding of the biological roles of microbial communities. To address this need, numerous computational approaches have been developed to infer functional potential and metabolic output from microbiome sequencing data, including PICRUSt2 and Tax4Fun [112,113]. These approaches differ substantially in terms of their underlying assumptions, resolution, data requirements, and interpretability [114].

### 6.1. Computational Inference of Functional Pathways and Metabolic Potential

#### 6.1.1. Marker Gene–Based Functional Inference

Tools such as PICRUSt2 and Tax4Fun are marker gene–based approaches designed to predict functional potential from 16S rRNA sequencing data [112,115]. In PICRUSt2, amplicon sequence variants are placed into a reference phylogenetic tree, and gene family abundances are inferred using ancestral state reconstruction before being mapped to functional pathways [112]. This approach relies on the assumption that phylogenetically related organisms share similar gene content. While this assumption is often valid at higher taxonomic levels, it may be violated due to strain-level variation, horizontal gene transfer, and incomplete representation of reference genomes. Consequently, 16S rRNA-based functional inference is best suited for identifying broad functional trends in well-characterized microbial communities and should be interpreted cautiously when strain-level resolution or precise pathway quantification is required [114].

#### 6.1.2. Read-Based Functional Profiling

Read-based approaches applied to shotgun metagenomic or metatranscriptomic data provide higher functional resolution than marker gene–based methods. HUMAnN and eggNOG-mapper are among the most widely used tools in this category [36,97]. HUMAnN employs a tiered alignment strategy in which sequencing reads are first mapped to species-specific pangenomes and subsequently to comprehensive protein databases such as UniRef. This is followed by gene family quantification and pathway reconstruction, enabling normalized, sample-to-sample quantitative comparisons [54].

These methods enable improved detection of strain-level functional variation and allow quantitative comparisons across samples; however, they require higher sequencing depth, increased computational resources, and more complex data preprocessing steps [78].

### 6.2. Genome-Scale Metabolic Models

Genome-scale metabolic models (GEMs) reconstruct organism-level metabolic networks from annotated genomes and integrate gene–protein–reaction relationships into constraint-based mathematical frameworks [116,117]. These models are commonly generated using tools such as the COBRA Toolbox, RAVEN, or CarveMe, which map genomic content to known biochemical reactions and simulate metabolic fluxes under defined constraints [118]. Community-scale GEMs extend this framework by combining species-specific models to enable the analysis of interspecies metabolic interactions and cross-feeding relationships [119]. Such approaches have been widely applied to investigate microbiome-associated metabolic perturbations in chronic diseases [120]. Nevertheless, most GEMs are stoichiometry-based and do not explicitly account for metabolite concentrations or kinetic parameters, which complicates the modeling of dynamic processes and reinforces reliance on steady-state assumptions. As a result, GEMs achieve maximal interpretive value when integrated with complementary experimental data and multi-omics measurements [121,122].

### 6.3. Metabolite Prediction Approaches

Metabolite prediction tools aim to infer metabolomic profiles directly from microbiome sequencing data, offering a cost-effective alternative to experimental metabolomics. Reference-based approaches estimate metabolite production by mapping gene or pathway abundances to biochemical databases such as KEGG, MetaCyc, or BioCyc. However, their performance is constrained by database coverage, particularly for uncultured or poorly characterized microorganisms [123,124]. Machine learning–based models, including MiMeNet and MelonnPan, attempt to overcome these limitations by learning gene–metabolite associations from large paired microbiome–metabolome datasets. While these approaches often achieve higher predictive accuracy, they are highly dependent on training data quality and typically lack mechanistic transparency, limiting biological interpretability and causal inference [125,126].

### 6.4. Methodological Dependencies and Causal Inference

Functional inference outcomes are strongly influenced by upstream bioinformatic steps, including sequence quality filtering, trimming, host DNA removal, assembly, gene prediction, and gene family assignment [116,127]. For example, insufficient removal of host-derived DNA can reduce effective microbial sequencing depth, whereas overly stringent filtering may introduce bias into gene abundance estimates [128]. Furthermore, the compositional nature of microbiome data must be considered when interpreting functional abundance profiles [129]. To move beyond correlation-based analyses, integrative frameworks combining constraint-based metabolic modeling with multi-omics data integration and statistical learning approaches have been proposed [130].

### 6.5. Strain-Level Variation, Functional Redundancy, and Database Effects

Strain-level genomic diversity can substantially alter enzyme repertoires and metabolic outputs even within the same species [131]. In addition, functional redundancy across taxa complicates pathway reconstruction, as similar metabolic functions may be encoded by multiple organisms [132]. Functional inference results are also highly dependent on the reference database used; KEGG, MetaCyc, UniRef, and eggNOG differ in terms of coverage and annotation depth [133,134].

The selection of analytical methods in microbiome studies should be guided by sample type, sequencing strategy, and the desired level of functional resolution. While 16S rRNA gene sequencing is suitable for identifying broad functional trends, detailed mechanistic and metabolic inference requires shotgun metagenomic and multi-omics approaches [59]. Visualization strategies such as pathway heatmaps, hierarchical pathway diagrams, and metabolic network maps facilitate the interpretation of high-dimensional functional data [135,136].

## 7. Statistical Analysis and Visualization

The selection of appropriate statistical analysis and visualization strategies is critical for addressing key biological questions in microbiome research, including community composition, between-group diversity differences, and identification of discriminatory taxa, genes, or functional pathways [137]. Given the high dimensionality, compositional nature, and sparsity of microbiome datasets, methodological choices at this stage can substantially influence both statistical validity and biological interpretation.

### 7.1. Statistical Methods for Bioinformatics Data

Descriptive statistics provide an essential first step for summarizing large and complex bioinformatics datasets by characterizing central tendency, dispersion, and data structure [138]. In microbiome and metagenomic analyses, these measures are commonly used to describe abundance distributions, diversity metrics, and sequencing coverage. While the mean offers a measure of central tendency, it is often sensitive to outliers and skewed distributions, which are prevalent in omics data. Consequently, robust statistics, such as the median and interquartile range (IQR), are generally preferred for asymmetrically distributed microbiome datasets [139]. Measures of variability, including variance and standard deviation, further provide insight into technical noise and biological heterogeneity within samples [140].

Inferential statistics enable extrapolation from observed data to broader biological populations by explicitly accounting for uncertainty and variability [141]. Hypothesis testing is widely used to assess whether observed differences between microbial communities, genes, or pathways reflect true biological variation rather than random sampling effects. The choice between parametric (e.g., *t*-test, ANOVA) and non-parametric tests (e.g., Mann–Whitney U, Kruskal–Wallis) should be guided by data distribution, sample size, and variance homogeneity [142,143]. Given the high dimensionality of microbiome data, multiple hypothesis testing correction, most commonly using the Benjamini–Hochberg false discovery rate (FDR), is essential to control type 1 error rates and ensure reproducible findings [144].

Regression-based approaches are increasingly applied to model relationships between microbial features and environmental, clinical, or experimental variables. Univariate regression models assess single-predictor-outcome relationships, whereas multivariate regression frameworks allow the simultaneous evaluation of multiple covariates and are particularly valuable in transcriptomic and metagenomic studies where confounding factors are common [145]. Additionally, multivariate exploratory techniques, such as principal coordinate analysis (PCoA), clustering, and factor analysis, facilitate the identification of latent structure, group separation, and compositional gradients within complex bioinformatics datasets [146].

### 7.2. Biodiversity Analysis

Alpha diversity quantifies microbial diversity within individual samples and is commonly assessed through measures of species richness and evenness. Richness reflects the number of distinct taxa present, whereas evenness captures the distribution of abundances across taxa. Among the most frequently used indices, the Simpson index emphasizes dominance patterns and decreases with increasing community diversity, often reported as its inverse (1–D) to enhance interpretability. In contrast, the Shannon index (H) captures both richness and evenness and increases with greater community balance, although it may be sensitive to sequencing depth and error rates [147]. Alpha diversity metrics can be calculated using tools such as QIIME, vegan (R), and USEARCH, and are typically visualized using box plots to facilitate group-wise comparisons [148,149]. Statistical differences between groups are commonly evaluated using ANOVA or non-parametric alternatives, depending on data distribution.

Beta diversity assesses compositional differences between samples and provides insight into community dissimilarity across experimental conditions. Compared to alpha diversity, beta diversity metrics are generally less sensitive to biases introduced during DNA extraction or PCR amplification. Commonly used distance measures include Bray–Curtis and Jaccard indices, which are non-phylogenetic, as well as UniFrac and UPGMA, which incorporate phylogenetic relationships among taxa [127]. While Jaccard distances consider presence–absence information only, Bray–Curtis accounts for relative abundances, and UniFrac further integrates evolutionary distances between taxa [150,151]. Statistical significance of beta diversity differences between groups is most commonly assessed using permutational multivariate analysis of variance (PERMANOVA) implemented via the adonis() function in the vegan package [152].

### 7.3. Statistical Software and Programming Environments

Among available computational platforms, R remains the most widely adopted language for statistical analysis and visualization in bioinformatics due to its extensive ecosystem of specialized packages. Tools such as ggplot2 and dplyr support high-quality visualization and efficient data manipulation, respectively [153]. Python, through libraries including SciPy, StatsModels, and pandas, is particularly advantageous for handling large-scale datasets and integrating machine learning workflows. To ensure reproducibility and dependency management, bioinformatics pipelines increasingly rely on environment managers such as Conda or its high-performance alternative Mamba, which facilitate consistent software deployment across R, Python, and command-line tools. Additionally, Bioconductor provides a comprehensive collection of statistical and analytical tools specifically tailored for genomic and transcriptomic data analysis [152]. While platforms such as SPSS, SAS, and MATLAB remain in use, their limited flexibility and extensibility often render them less suitable for modern microbiome bioinformatics pipelines.

### 7.4. Data Visualization Techniques

Effective data visualization is indispensable for interpreting microbiome data and communicating complex statistical results. Alpha diversity metrics are commonly visualized using box plots to highlight within- and between-group variability. Beta diversity analyses are frequently coupled with dimensionality reduction techniques such as PCoA, non-metric multidimensional scaling (NMDS), or constrained PCoA (CPCoA) to generate low-dimensional representations of community dissimilarity, typically visualized as scatter plots.

Taxonomic composition is most often displayed using stacked bar charts, commonly aggregated at the phylum or genus level to enhance interpretability. To identify taxa, genes, or pathways that differ significantly between experimental groups, differential abundance analyses are performed using statistical tests such as Welch’s *t*-test, Mann–Whitney U test, or Kruskal–Wallis test, as well as specialized tools including ALDEx2, edgeR, and STAMP [154,155]. Results from these analyses are frequently visualized using volcano plots, Manhattan plots, or error bar plots, which collectively facilitate the identification of biologically meaningful biomarkers while accounting for statistical significance and effect size [4,76].

## 8. Technical and Biological Biases in Microbiome Research

Microbiome research is subject to multiple sources of bias arising across the analytical workflow, from sample collection to data interpretation. To improve clarity, these biases should be systematically classified with important implications for reproducibility and cross-study comparability. Biases in microbiome research are cumulative, as methodological errors introduced during sampling, extraction, sequencing, or bioinformatic processing can propagate across the workflow and collectively distort final analytical outcomes [78].

### 8.1. Sampling and Ecological Sources of Biases

Patient selection is critically important in microbiome studies to prevent biological biases. Since variables such as age, sex, ethnicity, diet, medication use, and lifestyle can influence the composition of the microbiome, the homogeneity of included individuals must be statistically controlled before initiating the study [149]. These sources of biological variation represent natural and biologically meaningful differences between individuals and should be distinguished from technical noise introduced during laboratory processing and sequencing, as they affect data interpretation in fundamentally different ways.

### 8.2. Extraction Related Biases

Sample-specific factors can introduce substantial extraction-related bias that directly affects the observed microbiome profile. Insufficient removal of host DNA may lead to a dominance of human genomic material and reducing sensitivity for low-abundance taxa. Elevated levels of host DNA can also increase sequencing costs while biasing both taxonomic profiling and functional inference by masking microbial diversity and functional potential. In addition, host contamination may skew relative abundance estimates by preferentially retaining microbial taxa that are more resistant to lysis or extraction procedures [55]. Furthermore, blood contamination in samples may lead to inhibition of downstream analyses and should be considered a potential source of extraction bias [69].

### 8.3. Library Preparation and Sequencing Biases

Library preparation is a major source of technical bias in microbiome sequencing studies, as molecular processes at this stage can alter the relative representation of taxa prior to sequencing. In amplicon-based analyses, primer–template mismatches may lead to preferential amplification of specific microbial groups, while PCR-related factors such as cycle number and GC-content–dependent amplification efficiency can further distort community composition. In addition, variation in adapter ligation efficiency during library construction may result in unequal incorporation of fragments into the sequencing library [78]. Together, these factors can propagate bias into downstream taxonomic and functional analyses, ultimately affecting diversity estimates and differential abundance results [76,152,153].

### 8.4. Bioinformatic and Database-Related Biases

Microbiome data are compositional rather than absolute, and the use of conventional normalization and standard statistical tests may therefore lead to misleading inferences. To reduce analytical bias, compositional data analysis approaches such as centered log-ratio (CLR) transformation are recommended, together with appropriate multiple-testing correction methods (e.g., FDR, Benjamini–Hochberg) [154,155,156]. Database-related bias represents another important source of distortion in microbiome studies, arising from outdated taxonomies, inconsistent nomenclature, incomplete genome catalogs, and uneven representation of microbial groups, which can result in misclassification and biased taxonomic and functional outputs. Transparent reporting of database names and versions and cautious interpretation of results are therefore essential to improve reproducibility and cross-study comparability [40].

### 8.5. Contamination Biases

Laboratory contamination, sample cross-contamination, index hopping, background DNA, and reagent-derived contaminants are well-recognized sources of bias in microbiome analyses, particularly in low-biomass samples where contaminant DNA may constitute a substantial proportion of sequencing reads. If not adequately controlled, such contamination can lead to false-positive taxonomic assignments, inflated diversity estimates, and misleading biological interpretations. The use of negative controls and sequencing blanks is therefore essential to identify non-biological microbial signatures, while comparative analyses with controls combined with bioinformatic filtering and visualization approaches enable contamination detection and mitigation [157,158]. In addition, host-DNA contamination poses a major challenge in host-associated microbiome studies by reducing microbial signal and masking low-abundance taxa, thereby biasing taxonomic and functional inference. Experimental host-DNA depletion strategies and bioinformatic removal of host-derived reads are commonly applied to improve sequencing efficiency, data accuracy, and reproducibility [40].

### 8.6. Batch Effects as a Source of Technical Bias

A batch refers to a group of samples processed together within the same time frame and under identical technical conditions. In microbiome research, batch effects describe systematic, non-biological variation introduced during sample processing, library preparation, storage, or sequencing that is unrelated to the biological variables of interest. Such effects commonly manifest as unexpected clustering in ordination analyses (e.g., PCA or PCoA), distortion of alpha and beta diversity metrics, and spurious differential abundance results. Detection typically relies on exploratory visualization approaches combined with the inclusion of control samples and technical replicates. Practical mitigation strategies include harmonization of experimental protocols, use of standardized reagents and consistent storage conditions, normalization and statistical batch-correction methods during data analysis. Failure to adequately address batch effects can compromise reproducibility and cross-study comparability [159]. These approaches represent practical strategies to reduce technical bias across microbiome workflows.

### 8.7. Bias Detection and Assessment Strategies

Bias detection in microbiome studies can be supported by the use of appropriate controls, mock communities, or spike-in standards, which provide reference benchmarks for evaluating deviations introduced during laboratory and analytical workflows. In addition, exploratory statistical approaches such as alpha- and beta-diversity metrics, principal component analysis (PCA), and PCoA are commonly used to identify unexpected clustering patterns indicative of technical variation rather than biological structure. Heatmap visualizations further facilitate the detection of systematic abundance shifts across samples, which may suggest batch effects or protocol-related bias [78].

Technical and biological biases pose major challenges to reproducibility in microbiome research, as methodological variation across studies can lead to inconsistent findings. While biological variation reflects genuine differences between populations or environments, unrecognized technical bias may obscure true biological signals and compromise cross-study comparability. Consequently, inadequate control of both sources of variability can hinder reproducibility, highlighting the need for standardized methodologies and transparent reporting [78].

### 8.8. Representative Examples Illustrating the Impact of Bias in Microbiome Studies

Representative studies have demonstrated that bias can substantially distort microbiome study findings. For example, batch effects may drive artificial sample clustering unrelated to biological conditions, while contamination in low-biomass samples can result in false-positive taxonomic assignments dominated by reagent-derived DNA. In addition, database-related limitations have been shown to bias taxonomic classification and functional interpretation. Together, these examples illustrate how unrecognized bias can generate spurious associations and compromise the validity and reproducibility of microbiome research [78].

## 9. Applications in Various Disciplines

Microbiome studies are important not only for human health but also for animal health, agricultural technology, and environmental biotechnology. By generalizing the concepts of the One Health Microbiome and One World–One Health, these studies aim to uncover the full potential of microbial ecosystems, offering new opportunities for innovation and sustainability [129,160]. For these applications to transition from research settings to clinical practice, regulatory frameworks, and industrial implementation, reproducibility and standardization are critical. Reproducible microbiome-based findings are essential for regulatory approval, clinical validation, and the scalability of industrial applications, particularly in food systems and therapeutics. Variability in sampling, sequencing platforms, bioinformatic pipelines, and data interpretation continues to pose a major barrier to translation.

Research on the microbiome, like any research field, requires great care. Many steps can affect the accuracy of results in microbiome studies. Careful planning of the study design, sample collection, sample storage conditions, processing, and analysis steps is crucial. During the analysis phase, many factors must be considered, including antibiotic use, age, gender, diet, and geographical factors. Sampling strategy: Microbial distribution in environmental samples can be affected by factors such as spatial and seasonal or intraday variations. The collected sample must be representative of the entire population. Therefore, standardizing the sampling strategy is important. During the sampling phase, there are steps that can affect the results, such as technical and analytical problems, and standardization issues. The microbial biomass within the sample is important. Some samples have low microbial biomass, and this affects the results. Sampling method, temporal and environmental variations, changes related to sample storage, management of environmental contamination, gene region selection and method selection are all factors that influence the results. Therefore, planning of all steps, standardization, and quality control studies are extremely important [161].

Microbiome studies in veterinary medicine are crucial for animal diseases and livestock research. They offer unique techniques to enhance productivity and reduce antibiotic use. Since soil microbiome affects plant health, crop resilience and productivity, microbiome research is critical for sustainable agriculture. Microbiomes contribute substantially to nutrient cycling, disease and insect pest suppression, stress resilience, phytohormone regulation, and food processing. Agricultural products derived from microbiome research can significantly enhance plant health and agricultural productivity, while simultaneously aiding in the prevention of animal diseases and improving nutrient utilization in humans. Such microbiome-based approaches may be crucial in addressing malnutrition and gut dysbiosis in populations affected by climate-driven displacement [130,160].

The microbiome plays a crucial role in environmental applications, including the control of water, air, and soil pollution. Recent decades have highlighted the potential of microorganisms, particularly bacteria, as effective agents for the remediation of soil, water, and air contaminants through their catalytic activities, offering sustainable alternatives to chemical-based approaches. Bacteria can remove a broad range of pollutants, including antibiotics, agrochemicals, radioactive elements, and petroleum-derived compounds. Moreover, biofiltration has emerged as a promising strategy for controlling industrial air pollution, with several bacterial species demonstrating efficacy in biofilter systems. Certain bacteria, such as *Acinetobacter, Bacillus, Pseudomonas*, and *Rhodococcus* spp., have also shown the ability to degrade microplastic and nanoplastic residues in soil via biodeterioration, biofragmentation, assimilation, and mineralization [162].

The microbiome has also emerged as a pivotal research theme within food systems due to its capacity to enhance food safety, promote sustainability, optimize production yields, and identify novel microbial strains, probiotics, and mobile genetic elements. A deeper understanding of microbial resources is facilitating precision management of food systems—not only in research but also in industrial applications. Several European initiatives, such as CIRCLES, HoloFood, MASTER, SIMBA, and MicrobiomeSupport, are investigating microbiome dynamics across the food supply chain, highlighting the growing recognition of microbiome-based innovations as essential contributors to the global economy. However, to maximize impact, the field must shift from predominantly observational studies toward more mechanistic explorations in food science, supported by reproducible multi-omics workflows and harmonized analytical frameworks [53].

Multi-omics approaches in food studies have so far been applied mainly to fermented dairy products, with increasing attention to meat and plant-based foods. In practice, the integration of metagenomics, metatranscriptomics, metabolomics, and proteomics enables pathway-level analysis of microbial functions, allowing researchers to link community composition with metabolic activity, host–microbe interactions, and functional outcomes such as flavor development, spoilage dynamics, or pathogen suppression. Combined datasets are increasingly used to predict microbial responses to environmental stressors, processing conditions, and antimicrobial interventions, as well as to support drug-response modeling and functional risk assessment. These efforts typically aim to map microbial populations throughout the food chain, identify rare or novel taxa and microbial adaptation strategies, correlate microbiome attributes with food quality and safety outcomes, translate microbiome data into practical industrial applications, and support microbial risk assessments [53].

The global demand for safe, nutritious foods with minimal synthetic additives is rising. The World Health Organization (2019) reports that about 600 million people suffer annually from foodborne diarrheal diseases, resulting in an estimated 420,000 deaths [163]. Because food undergoes multiple stages of processing before consumption, the role of microbiome—whether in fermentation or spoilage—is crucial. Bioinformatics, which leverages computational models to interpret biological data, has become indispensable in food and nutritional sciences, enabling the identification of functional genes, proteins, and metabolites involved in key biological processes. Importantly, methodological choices—such as DNA extraction protocols, sequencing depth, reference databases, and statistical models—can significantly influence application outcomes. For example, differences in bioinformatic pipelines may lead to contrasting conclusions in clinical diagnostics, environmental monitoring, or industrial microbiome optimization, underscoring the need for transparent and standardized analytical strategies [163].

Despite advances in multi-omics, amplicon-based sequencing of 16S rRNA remains a cornerstone method for microbial profiling in food systems, especially for pathogen detection and understanding microbial roles during fermentation. Importantly, interactions between microbial communities and their surrounding ecosystems strongly influence fermentation efficiency. Case studies in fermented food production and environmental monitoring have demonstrated that integrating amplicon data with metabolomic or functional genomic analyses improves predictive accuracy and supports more robust industrial decision-making.

### 9.1. Microbiome Engineering

The Human Microbiome Project (HMP) has contributed to the development of microbiome engineering by providing an understanding of the characteristics of healthy and unhealthy microbiome, particularly in the gut, mouth, skin, and urogenital regions. Microbiome engineering is most widely applied to the human microbiome [164].

Ecosystem structure and function are largely shaped by their core microbiomes. Microbiome engineering seeks to alter microbial community structures and restore ecological balance. Strategies include modifying microbiome dynamics with probiotics or prebiotics, modulating functionality via DNA conjugation-mediated engineering or enzyme inhibitors, and developing therapeutic applications using natural or synthetic microbial consortia, such as fecal microbiome transplantation (FMT) and fecal virome transplantation [129,165,166].

Gut microbiome imbalance (dysbiosis) is thought to be important in the pathogenesis of intestinal disorders such as inflammatory bowel disease and irritable bowel syndrome, as well as extraintestinal disorders such as allergies, type 1 diabetes, cardiovascular disease, metabolic syndrome, and obesity [167]. Studies have shown that prebiotic inulin or inulin-type fructans modulate the colonic microbiome and have revealed significant increases in *Faecalibacterium prausnitzii* and two *Bifidobacterium* spp., *B. adolescentis* and *B. bifidum* [168]. Prebiotics have been shown to reduce allergic reactions and infections in infancy. Formula milk supplemented with a prebiotic blend of galacto-oligosaccharides (GOS) and long-chain inulin has been shown to significantly reduce the incidence of atopic dermatitis in infants with a parental history of atopy [169,170].

Emerging approaches also include the design of synthetic microbiomes with defined functional traits, the use of machine-learning models to identify predictive microbial biomarkers, and the development of microbiome-based therapeutics tailored to individual hosts. These microbial networks interact not only with one another but also with their hosts, responding dynamically to the metabolites they generate [171]. Disruption of this balance can negatively affect host vitality and soil fertility. By engineering microbial communities, researchers can enhance host traits or support ecosystem health. Recent advances integrating artificial intelligence and multi-omics data have enabled the identification of functional signatures associated with disease resistance, nutrient utilization, and metabolic efficiency, paving the way for personalized nutrition and precision microbiome interventions. Although evidence suggests that microbiome engineering holds promise for disease treatment and agricultural improvement, the field remains in its infancy and requires rigorous validation and reproducible methodologies.

### 9.2. Challenges in Clinical Application

Despite rapid progress, the clinical application of microbiome science faces several challenges. Biologically, establishing causal links with the gut microbiome is difficult because of its heterogeneity and complexity. Methodologically, variability in diet, medications, and environmental factors, along with the lack of standardized protocols, hinders reproducibility. Logistically, personalized microbiome-based interventions remain difficult to implement, while regulatory uncertainty further complicates clinical translation. Culturally, skepticism among many clinicians continues to limit adoption in practice.

Fecal microbiome transplantation (FMT) is one established application, used for recurrent *Clostridium difficile* infection. Studies show a significant increase in deficient *Bacteroidetes* spp. after treatment [172]. FMT is typically performed via colonoscopy. To minimize complications, donor stool must be collected from a healthy individual after a full medical history review and blood testing [173]. However, variations in donor selection, preparation methods, and administration routes have been shown to influence therapeutic outcomes, highlighting how methodological decisions directly affect clinical efficacy and safety. These examples underscore the importance of standardized, reproducible frameworks for broader clinical and industrial adoption of microbiome-based therapies.

## 10. Future Perspectives and Technological Innovations

While major technological advances have expanded the analytical scope of microbiome research, the field continues to be dominated by exploratory and associative studies. To advance microbiome science toward reproducible, translatable, and clinically actionable outcomes, future research must adopt explicit, stepwise methodological and analytical strategies rather than relying on broad technological trends alone.

A critical next step in microbiome research is the shift from descriptive profiling to validated, application-oriented workflows. This can be achieved by locking down pre-analytical and analytical pipelines through fixed protocols for sampling, nucleic acid extraction, library preparation, and sequencing depth, together with predefined quality-control thresholds, thereby reducing inter-study and inter-laboratory variability [1,9,78]. Second, microbiome-derived biomarkers should be evaluated using independent validation cohorts rather than discovery datasets alone. Performance metrics such as sensitivity, specificity, robustness across sequencing platforms, and temporal stability must be reported systematically [4,7]. Third, analytical outputs should be aligned with clinically meaningful endpoints, enabling microbiome features to be assessed within diagnostic or prognostic frameworks. Together, these steps provide a practical pathway for translating microbiome research into clinical-grade applications without requiring immediate regulatory approval [5,172,173].

Future microbiome studies must move beyond general calls for standardization and implement operationally defined benchmarking strategies. This includes the routine use of mock microbial communities, synthetic reference datasets, and negative controls across all stages of analysis to quantify technical bias and analytical accuracy [44,45].

Equally important is the use of structured metadata schemas capturing key host, environmental, and technical variables. Harmonized metadata enables cross-cohort comparisons and meta-analyses essential for biomarker validation and clinical translation, whereas the absence of enforceable standards continues to limit reproducibility across studies [1,9].

Artificial intelligence and machine learning should be integrated into microbiome research at clearly defined analytical stages, rather than applied as exploratory tools. In the near term, supervised machine learning models such as random forests and gradient boosting should be prioritized for biomarker discovery and outcome prediction, while deep learning architectures are better suited for integrating longitudinal multi-omics data and complex host metadata [7,109]. To ensure clinical relevance, AI-derived features must be validated across independent cohorts and implemented in version-controlled analytical pipelines. Model interpretability and transparent performance reporting are essential to prevent overfitting and support biological plausibility, enabling AI-based analyses to progress from exploratory tools to reproducible components of microbiome-based diagnostics [111].

Emerging sequencing technologies should be evaluated based on their practical contribution to resolution and interpretability, rather than novelty alone. Ultra-long read sequencing and adaptive sampling can be strategically applied to resolve strain-level variation, mobile genetic elements, and antimicrobial resistance determinants that are poorly captured by short-read approaches [45,80]. Single-cell metagenomics and Hi-C–based binning should be incorporated selectively to link plasmids, phages, and accessory genes to host genomes, particularly in studies focusing on horizontal gene transfer and microbial ecology [4,79]. Future studies should explicitly define which biological questions require these high-resolution methods, thereby optimizing cost-effectiveness and analytical clarity. As microbiome datasets continue to grow in size and complexity, future research must adopt scalable computational infrastructures capable of supporting multi-omics integration and longitudinal analyses. High-performance computing and cloud-based platforms should be combined with automated workflow management systems to enable reproducible and efficient data processing [68,111].

The routine use of containerized, version-controlled pipelines will be essential for ensuring analytical transparency, cross-study comparability, and regulatory readiness [80]. Investment in computational standardization is therefore not ancillary but central to the future viability of microbiome research. Future multi-omics studies should focus on joint modeling strategies that explicitly integrate metagenomic, metatranscriptomic, proteomic, and metabolomic data rather than analyzing each layer independently. Genome-scale metabolic models and network-based approaches provide a practical framework for translating multi-omics data into testable mechanistic hypotheses [106,110]. To achieve this, harmonized preprocessing pipelines and batch-correction strategies must be defined a priori. Such structured integration will allow functional validation of taxonomic signals and improve causal inference in host–microbiome interactions [89,90].

The expansion of microbiome research into personalized medicine, nutrition, and agriculture should be guided by application-driven study designs. In clinical contexts, microbiome-informed dietary or therapeutic interventions should be evaluated using standardized outcome measures and longitudinal monitoring [107].

In agricultural systems, microbiome-based strategies should prioritize reproducibility under field conditions and measurable impacts on productivity and sustainability [53,129,160]. Defining application-specific performance criteria will be essential for translating microbiome insights into real-world solutions. Long-term advancement of microbiome research requires explicit integration of ecological and evolutionary frameworks. Microbial competition, cooperation, horizontal gene transfer, and phage–microbe interactions directly influence community stability and functional resilience [4,105]. Future studies should incorporate phageomics and evolutionary modeling to predict community responses to environmental or therapeutic perturbations. Such approaches will enhance the interpretability and durability of microbiome-based interventions. As microbiome research approaches clinical and commercial deployment, ethical and regulatory considerations must be addressed proactively. Standardized policies for data governance, privacy protection, and informed consent are essential for responsible data use [1,5]. Furthermore, the underrepresentation of diverse populations in microbiome datasets must be corrected through inclusive study designs. Addressing these issues will be critical for ensuring equitable access to microbiome-based technologies and preventing population-specific bias.

Key open questions for the field include the causal attribution of microbial functions to disease phenotypes, the long-term stability of microbiome-derived biomarkers, and the safety of microbiome-targeted interventions [4,7]. Addressing these challenges requires coordinated, hypothesis-driven research supported by standardized methodologies.

In low- and middle-income regions, future efforts should prioritize field-adapted sampling strategies, cost-effective sequencing technologies, and region-specific reference databases to ensure global representation in microbiome research [17,129]. Moreover, future studies should move beyond descriptive association analyses and be guided by clearly defined, falsifiable hypotheses supported by controlled or longitudinal study designs [4,7]. Concrete next steps include defining causal, testable hypotheses linking specific microbial taxa, genes, or metabolic pathways to host phenotypes and evaluating them through perturbation-based or longitudinal approaches, such as dietary interventions or time-resolved sampling [90,91]. In parallel, studies should predefine analytical endpoints and success criteria to ensure reproducibility and biological relevance across cohorts [79]. Such hypothesis-driven frameworks are critical for advancing microbiome research beyond descriptive analyses. In microbiome research, the fact that existing reference catalogs are largely derived from high-income populations limits the generalizability of microbiome-based biomarkers and functional inferences [17,105]. Future research should prioritize field-adapted sampling, low-cost portable sequencing, and simplified bioinformatic workflows for resource-limited settings, alongside region-specific reference databases that reflect local diets, environmental exposures, and host genetics. Addressing these gaps will enhance equity and improve the robustness and global applicability of microbiome-based diagnostics and interventions [17,80,105,129].

## 11. Conclusions

Microbiome research has become a common area of interest not only in human–animal studies but also in multidisciplinary fields such as agriculture, food science, and the environment. Advances in sequencing technologies and bioinformatics tools have enabled us to characterize microbial diversity, elucidate host-microbe interactions, translate findings into clinical and industrial applications, and even model future scenarios using machine learning.

Looking ahead, a shift from descriptive studies to mechanistic research is inevitable to unlock the full potential of the microbiome. Microbiome engineering, synthetic biology, and precision interventions offer promising avenues for therapeutic innovation and sustainable food systems. At the same time, ensuring ethical practices, data compliance, and interdisciplinary collaboration is crucial for the responsible advancement of this field. Ultimately, a deeper understanding and application of microbial ecosystems has the potential to transform global health, food security, and environmental sustainability under the One Health framework.

## Figures and Tables

**Table 1:** Comparison of commonly used sample collection methods in gastrointestinal microbiota research, highlighting the anatomical regions represented, their analytical applications, and key advantages and limitations.

Sample Collection Method (Reference)	Represented Region/Target of Analysis	Advantages	Disadvantages/Limitations
Stool sample [9,12]	Colonic luminal microbiota and metabolites (SCFAs)	Gold standard; high microbial diversity; suitable for compositional and metabolomic analyses	Patient compliance may be low; heterogeneous structure; sensitive to collection and storage conditions
Rectal swab [9,11,12]	Distal colonic luminal microbiota	Non-invasive; applicable when stool samples cannot be obtained; rapid and practical	Limited suitability for metabolite analyses; low sample biomass; microbial composition may change when stored at room temperature
ESwab rectal swab [11,12,13]	Distal colonic microbiota	Standardized transport medium; comparable to stool in terms of alpha diversity	Increased *Escherichia coli* abundance may occur during room temperature storage; not suitable for SCFA analysis
Glove-tip sampling [9]	Rectal canal and distal colonic lumen	Applicable in intensive care and unconscious patients; non-invasive	Lack of standardized protocols; risk of contamination; low sample volume
Catheter- or endoscopic-based sampling and mucosal biopsy [10]	Specific colonic segments and mucosa-associated microbiota	Enables region-specific and mucosa-associated microbiota analysis; directly reflects host–microbiota interactions	Invasive; requires clinical procedures; ethical and logistical constraints; does not fully represent luminal microbiota; not suitable for routine microbiome studies
Smart toilet/automated collection systems [16]	Contamination-free stool	Enables urine–feces separation; potential for standardization	Limited accessibility; high cost

**Table 2:** Key pre-analytical variables influencing gastrointestinal microbiota research, including participant-related factors, sampling strategies, and sample handling and storage conditions that may affect microbial composition, diversity, and data comparability.

Pre-Analytical Variable (Reference)	Impact on the Microbiota	Recommendations/Considerations
Study design [16,20]	Introduces methodological variability and limits comparability	Standardized protocols should be used and all steps should be reported in detail
Participant characteristics and medication use [14,15]	Significantly influences microbial diversity and composition	Age, sex, genetic background, immune status, diet, and antibiotic/PPI use should be documented
Sample type [16,20]	Determines the representativeness of microbial composition	Stool is the gold standard; rectal swabs may serve as an appropriate alternative
Sampling method [9,10]	Affects contamination risk and sample quantity	Standardized and validated collection methods should be preferred
Sampling time [12]	Diurnal variation alters microbiota profiles	The first complete bowel movement of the day is recommended
Stool consistency/intestinal transit time [16,18]	Reduced species richness is observed in liquid stools	Stool consistency should be reported using the Bristol Stool Scale
Homogenization [16,20,21]	Taxonomic differences occur between inner and outer stool regions	Homogenization should be performed prior to analysis
Aliquoting and freeze–thaw cycles [16,20]	Repeated freezing and thawing compromise DNA integrity and microbial profiles	Samples should be aliquoted; freeze–thaw cycles should be limited to ≤3
Transport duration and temperature [16,23]	Prolonged transport and inappropriate temperatures lead to microbial shifts	≤4 h at room temperature or ≤24 h at 4 °C
Use of preservatives [16,23]	Affects DNA stability and microbial viability	Preservatives should be selected according to study objectives
Storage temperature [16,23]	Determines long-term microbiota stability	−20 °C for short-term and −80 °C for long-term storage

**Table 3:** Preanalytical steps and key components in skin microbiome research [24,25,26,27,28].

Step	Preanalytical Process Component	Description/Examples
Step 1	Selection of the skin site for sampling	Sebaceous, moist, or dry skin sites; presence or absence of dermal disease and disease stage
Step 2	Identification of participant-related variables	Age, sex, ethnicity, and presence of underlying diseases
Step 3	Selection of the sampling method	Superficial swab, tape stripping, skin scraping, punch biopsy
Step 4	Determination of sample storage conditions	Storage at −80 °C; use of ethanol or similar preservative solutions

**Table 4:** Sampling methods used in respiratory microbiome research and their main characteristics [34].

Sample Type	Represented Region	Advantages	Disadvantages/Limitations	Key Features
Nasal/Nasopharyngeal swab	Upper respiratory tract	Easy to apply, non-invasive, repeatable	Does not represent the lower respiratory tract	Low cost, suitable for screening studies
Sputum	Upper + lower respiratory tract	Non-invasive, high microbial biomass	Risk of oropharyngeal contamination, limited anatomical specificity	Widely used in clinical practice
Tracheal aspirate	Upper + lower respiratory tract	Microbial profile comparable to BAL, relatively less invasive	Risk of contamination, unclear anatomical origin	Frequently used in intensive care patients
Bronchoalveolar lavage (BAL)	Lower respiratory tract/alveoli	Better representation of the lower respiratory tract	Invasive, low biomass, risk of contamination	Reference method for lung microbiome studies
Protected brush specimen	Lower respiratory tract	Low risk of contamination	Highly invasive, technical complexity	Enables site-specific sampling
Lung biopsy	Lung parenchyma	Tissue-specific, highest anatomical accuracy	Highly invasive, ethical and clinical limitations	Only method for parenchymal microbiota analysis

**Table 5:** Impact of lysis strategies on different microbial groups and potential sources of bias in microbiome studies.

Microbial Group	Cell Wall Structure	Preferred Lysis Strategy	Potential Bias if Lysis Is Insufficient
Gram-negative bacteria	Thin peptidoglycan layer with outer membrane	Chemical (sodium dodecyl sulfate (SDS) or cetyltrimethylammonium bromide (CTAB)) and/or enzymatic lysis (proteinase K)	Overrepresentation when mechanical lysis is omitted
Gram-positive bacteria	Thick peptidoglycan layer	Mechanical lysis (bead beating) ± enzymatic	Underrepresentation of Bacillota and Actinomycetota
*Mycobacteria*	Mycolic acid–rich outer membrane	Intensive mechanical lysis combined with chemical treatment	Severe underrepresentation or false negatives
Spore-forming bacteria	Multilayered, highly resistant structures	Prolonged or intensified mechanical disruption	Failure to detect dormant or resistant taxa
Fungi	Chitin- and glucan-rich cell wall	Mechanical lysis (bead beating/liquid nitrogen grinding)	Underestimation of fungal diversity
Low-biomass samples	High host DNA content, low microbial load	Gentle lysis with pre-treatment steps	Contamination-driven distortion of microbial profiles

**Table 6:** Considerations for selecting nucleic acid extraction kits based on sample biomass and analytical targets.

Sample Type/Study Aim	Key Challenges	Recommended Extraction Features	Rationale	Representative References
High-biomass samples (e.g., adult feces)	High microbial load, complex matrices	Strong mechanical lysis, broad-spectrum DNA recovery	Maximizes recovery of Gram-positive and structurally robust taxa	[52]
Low-biomass samples (e.g., lung tissue, skin, placenta)	Low microbial DNA, high contamination risk	Low-contaminant kits, inclusion of extraction blanks, host DNA reduction	Minimizes reagent-derived background signals	[42,53]
DNA-based microbiome studies	Taxonomic profiling, relative abundance	Efficient lysis with minimal taxonomic bias	Preserves representative community structure	[40]
RNA-based microbiome studies	RNA instability, RNase activity	Rapid stabilization, RNase-free reagents, optimized RNA chemistry	Captures transcriptionally active microbiome	[54]
Host-rich samples	Excess host nucleic acids	Host DNA depletion or selective microbial lysis	Improves microbial signal-to-noise ratio	[55]

**Table 7:** Comparative benchmarking studies (2020–2025) evaluating nucleic acid extraction kits and protocols in microbiome research.

Year	Sample Type	Extraction Kits/Protocols Compared	Main Findings	Relevance for Microbiome Studies	References
2020	Respiratory	two automated platforms (eMAG); MagNA Pure 24 and MP24 vs. manual QIAamp Viral RNA Mini Kit	The QIAamp method produced a reduced percentage of viral readings for both clinical and mock samples. The sample cross-contamination was elevated with MP24. Potential reagent contamination was detected.	Selecting appropriate extraction strategy is vital for precise virome characterization.	[62]
2021	Skin, stool, urine, tissue, oral specimens, soil, water, fermented food	MagAttract PowerSoil DNA isolation kit vs. the MagMAX microbiome ultra nucleic acid isolation kit	The DNA yield was comparable among the three extraction procedures.	Robust connections in microbial community beta-diversity among specimens across the extraction techniques; the associations with the PowerSoil protocol were better for MagMAX 2 min vs. for MagMAX 20 min.	[51]
2021	Breast milk	Qiagen MagAttract Microbial DNA Isolation Kit vs. Norgen Milk Bacterial DNA Isolation Kit vs. Qiagen MagAttract Microbiome DNA/RNA Isolation Kit vs. TRIzol LS Reagent	The QM was the most appropriate kit for the extraction of bacterial DNA from human milk.	The selection of the extraction method influences the efficacy of bacterial DNA yield from human milk and the subsequent bacterial community profiles derived.	[63]
2022	Cervicovaginal	QIAamp DNA Microbiome Kit vs. DNeasy Blood & Tissue kit with enzymatic pre-treatment for improved lysis of Gram-positive bacteria	The extraction of DNA from cervicovaginal materials utilizing the DNeasy Blood and Tissue kit, following pretreatment with lysozyme and mutanolysin, yielded superior DNA quantities, enhanced bacterial diversity, and improved species representation relative to the QIAamp DNA Microbiome kit.	Employing the non-microbiome specific kit with an additional enzymatic pre-treatment yields better DNA yield, bacterial diversity, and representativeness relative to the more labor-intensive microbiome-specific DNA extraction kit, while both methods exhibit comparable low host coverage.	[64]
2022	Bile, stool, saliva, plaque, sputum, and conjunctival swab samples (Nanopore sequencing)	Qiagen DNeasy PowerSoil Pro vs. Qiagen QiAamp DNA Microbiome Kit vs. ZymoBIOMICS DNA Miniprep Kit	The variability introduced by the kits was minimal in comparison to the differences among sample types.	The study demonstrates early evidence of extraction-induced compositional bias	[65]
2023	Stool	Chemical and mechanical (bead beating) lysis vs. initial chemical and mechanical lysis with Maxwell^®^ RSC Faecal Microbiome DNA kit vs. Maxwell^®^ RSC Faecal Microbiome DNA kit + bead-beating vs. Maxwell^®^ RSC Faecal Microbiome DNA kit	Abundance of the Bacillota was lower; abundance of the *Bacteroidetes* and *Proteobacteria* were higher when DNA extraction without additional chemical and mechanical lysis used	Methodological standardization is necessary.	[66]
2024	Stool	QIAamp^®^ Fast DNA Stool Mini Kit vs. DNeasy PowerSoil Pro Kit vs. the International Human Microbiome Standards Protocol Q extraction method	DNeasy PowerSoil Pro Kit and Mini-Beadbeater-16 provided the better results than the manuel method.	A standard operating method for DNA extraction from human stool samples for mycobiome analysis were proposed.	[67]
2024	Stool	AllPrep DNA/RNA Mini Kit vs. QIAamp Fast DNA Stool Mini Kit	Different extraction methods alters read depth, DNA concentration, and DNA quality.	The method of DNA extraction substantially influences gut microbial diversity. The composition of microbial communities varies according to the DNA extraction method	[68]
2025	Stool from IBD patients	Qiagen DNEasy PowerSoil Pro vs. FastDNA Spin Kit for Soil	Various DNA extraction kits provided minimal influence on gut microbiome profile when other methodological factors have been addressed.	Comprehending the impact of methodological variability on microbiome compositions will, diminish research redundancy and enhance the universality of findings.	[69]

**Table 8:** Comparison of Major Sequencing Technologies Used in Microbiome Research.

Sequencing Approach/Platform	Advantages	Limitations	Reference
Sanger (1st Gen.)	High accuracy (~99.9%); Long read length (~1000 bp)	Low throughput; High cost; Limited application in microbiome analysis	[68]
16S rRNA amplicon workflow (Illumina-based)	Cost-effective and fast; High sample throughput; Extensive database support	Limited species-level resolution; PCR bias; Targets only specific gene regions	[75,76]
Shotgun metagenomic workflow	Species- and gene-level resolution; Enables functional analysis	High cost; Large data volume; Requires advanced computational infrastructure	[77,78]
Long-read platform (PacBio HiFi)	Long reads (15–25 kb); High accuracy; Complete 16S or genome sequencing	Expensive; Complex library preparation; Time-consuming data processing	[68,79]
Long-read platform (Oxford Nanopore)	Portable devices (e.g., MinION); Real-time data generation; Long reads	High error rate (5–15%); Software infrastructure still evolving	[71,80]
Single-cell sequencing workflows (Microbe-seq/M3-seq)	Single-cell genome and transcriptome resolution; Strain-level differentiation	High cost; Requires microfluidic infrastructure; Emerging technology	[68]
Minimal-input profiling approach (2bRAD-M)	High accuracy in low-biomass samples; Covers all microbial domains	Limited reference database; Few validation studies; Relatively new approach	[81]

**Table 9:** Types of bioinformatics analyses and corresponding software/tools.

Analysis Type	Description	Typical Input Data	Common Tools/Software	Main Applications
Genome Alignment	Mapping sequencing reads to a reference genome for comparative analysis and variant detection	FASTQ, BAM	BWA, Bowtie2, SAMtools	Genome annotation, variant detection, genetic disease research
Variant Calling	Identification of single nucleotide polymorphisms (SNPs), insertions/deletions (indels), and structural variants	BAM, VCF	GATK, FreeBayes, bcftools	Population genetics, mutation analysis, cancer genomics
RNA-seq Analysis	Quantitative analysis of gene expression and differential expression profiling	FASTQ, count matrix	STAR, HISAT2, DESeq2, edgeR	Gene expression comparison, disease biomarker discovery
Metagenomic Classification	Taxonomic profiling and estimation of microbial community composition	FASTQ, contigs, classification reports	Emu, Kraken2, MetaPhlAn3, Kaiju	Microbiome analysis, environmental microbiology, human gut studies
Functional Annotation	Assignment of biological functions to genes or proteins based on sequence similarity	FASTA, GFF, protein sequences	Prokka, eggNOG-mapper, InterProScan, SKESA	Genome function analysis, evolutionary and comparative genomics
Phylogenetic Analysis	Inference of evolutionary relationships and construction of phylogenetic trees	FASTA, multiple sequence alignments (MSA)	Clustal Omega, MAFFT, IQ-TREE, RAxML	Phylogeny reconstruction, species classification, evolutionary biology
Protein Structure Prediction	Computational prediction of three-dimensional protein structures from amino acid sequences	FASTA	AlphaFold, SWISS-MODEL, I-TASSER	Drug design, molecular biology, protein function analysis
Network Analysis	Modeling and analysis of gene–gene or protein–protein interaction networks	Network tables, interaction matrices	Cytoscape, STRING, BioGRID	Systems biology, pathway analysis, disease network modeling
Epigenomic Analysis	Analysis of epigenetic modifications such as DNA methylation and histone marks	FASTQ, BED	Bismark, MACS2, DeepTools	Gene regulation studies, developmental biology, cancer epigenetics
Machine Learning Applications	Pattern recognition, classification, and predictive modeling in biological datasets	Numerical matrices, feature tables	scikit-learn, TensorFlow, XGBoost, caret	Biomarker discovery, disease classification, treatment response prediction
Single-cell Analysis (scRNA-seq)	Gene expression profiling at single-cell resolution	FASTQ, UMI count matrices	Seurat, Scanpy, CellRanger	Cellular heterogeneity analysis, immunology, developmental biology
Multi-omics Integration	Integrated analysis of multiple omics layers (genomics, transcriptomics, proteomics, metabolomics)	Multi-layer omics datasets	MOFA+, mixOmics, OmicsIntegrator	Systems biology, multi-layer disease mechanism analysis

**Table 10:** Overview of databases and their integrated tools commonly employed in bioinformatic workflows. * OA: Open access.

Database	Data Type	Update Frequency	Target Group	Maintaining Institution	Primary Use	Integrated Tools	Accessibility
RefSeq	Genome/Protein	Frequent	All organisms	NCBI	General taxonomy	Kraken2, Kaiju, MEGAN	OA *
GTDB	Genome	Frequent	Bacteria/Archaea	GTDB Project	Phylogenetic classification	GTDB-Tk	OA
SILVA	rRNA	Frequent	Bacteria, Eukaryotes	Max Planck Institute	Amplicon-based analysis	QIIME, mothur	OA
Greengenes	rRNA	Relatively infrequent	Bacteria	LANL	Microbiome studies	QIIME	OA
MetaPhlAn	Marker genes	Frequent	Bacteria/Archaea	Harvard Biobakery	Species-level profiling	MetaPhlAn, HUMAnN	OA
Kaiju DB	Protein	Frequent	All organisms	University of Tübingen	Protein-based classification	Kaiju	OA
MGnify	Mixed (Genome/Protein)	Frequent	All organisms	EMBL-EBI	Taxonomic + functional analysis	MGnify Portal	OA

## Data Availability

No new data were created or analyzed in this study.
